# Concomitant existence of pheochromocytoma in a patient with multiple endocrine neoplasia type 1

**DOI:** 10.1186/s40792-016-0214-x

**Published:** 2016-08-30

**Authors:** Ryo Okada, Tatsuo Shimura, Shigeyuki Tsukida, Jin Ando, Yasuhide Kofunato, Tomoyuki Momma, Rei Yashima, Yoshihisa Koyama, Shinichi Suzuki, Seiichi Takenoshita

**Affiliations:** 1 Department of Organ Regulatory Surgery, Fukushima Medical University, Fukushima, Japan; 2 Department of Surgery, Ohara General Hospital, Fukushima, Japan; 3 Department of Thyroid and Endocrinology, Fukushima Medical University, Fukushima, Japan

**Keywords:** Multiple endocrine neoplasia 1, Pheochromocytoma, Giant insulinoma

## Abstract

**Background:**

Multiple endocrine neoplasia type 1 (MEN1) is an autosomal-dominant inherited disorder that is classically characterized by the presence of neoplastic lesions of the parathyroid glands, the anterior pituitary gland, and the pancreas. However, MEN1 with concomitant pheochromocytoma is extremely rare.

**Case report:**

We report a case of MEN1 concomitant with pheochromocytoma. A 44-year-old Japanese man, who had undergone total parathyroidectomy due to primary hyperparathyroidism at the age of 18, was referred to our hospital with a complaint of a large abdominal tumor. He was diagnosed as having a giant insulinoma (maximum diameter 18 cm) in the pancreatic tail, five other non-functional neuroendocrine tumors in the pancreatic body and tail, multiple liver metastases of pancreatic neuroendocrine tumors, a pituitary prolactinoma, non-functional adrenal cortical adenomas, a pheochromocytoma in addition to a subcutaneous neurofibroma, and a cutaneous fibroma. The genetic screening revealed a deletion mutation at codons 83–84 in exon 2 of the *MEN1* gene. He underwent distal pancreatectomy, splenectomy, cholecystectomy, right adrenalectomy, abdominal subcutaneous tumor excision, and cutaneous tumor biopsy for the purpose of tumor volume reduction. Extended right posterior segmentectomy with partial hepatectomy of S2, S3, and S8 was performed to resect residual tumors 9 months after the initial surgery. Although a newly formed liver metastasis was found 19 months after the hepatectomy, he is still alive 4 years and 4 months after the initial surgery.

**Conclusions:**

We reported an extremely rare case of giant insulinoma and simultaneous occurrence of pheochromocytoma and adrenal cortical adenoma in the ipsilateral adrenal gland in a patient clinically and genetically diagnosed as having MEN1.

## Background

Multiple endocrine neoplasia type 1 (MEN1) is an autosomal-dominant inherited disorder that is classically characterized by the presence of neoplastic lesions of the parathyroid glands, the anterior pituitary gland, and the pancreas [1–4]. Furthermore, multiple benign and malignant tumors occur in regions such as the adrenal cortex, the thymus, the bronchi, and the skin. The *MEN1* gene was identified in 1997 and is located on chromosome 11q13 encoding a 610-amino acid protein, *MENIN* [2]. Insulinoma, one of the pancreatic neuroendocrine tumors (NETs) of MEN1, causes obvious symptoms even small in size due to hypoglycemia and is usually found small (less than 2.0 cm) at diagnosis [5]. An incidence of adrenal involvement in patients with MEN1 is about 20 % [4], most of which lesions are derived from the adrenal cortex [4].

We report herein a case of MEN1 with a giant insulinoma of the pancreatic tail, non-functional adrenal cortical adenomas, and an ipsilateral pheochromocytoma.

## Case presentation

**Fig. 1:**
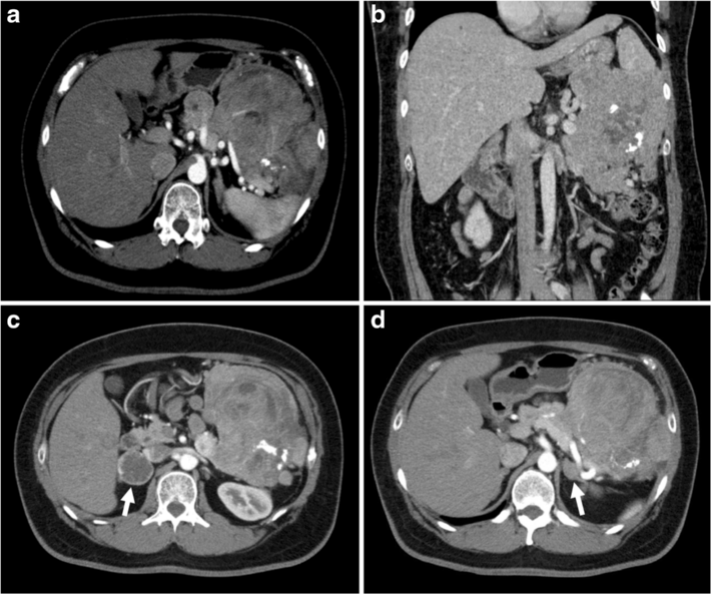
**a**, **b** Computed tomographic image of the abdomen, demonstrating a large was heterogeneously enhanced insulinoma arising from the tail of the pancreas. **c** Computed tomographic image of the abdomen, demonstrating a ring-enhanced tumor of the right adrenal gland (*arrow*). **d** Computed tomographic image of the abdomen, demonstrating a poor-enhanced tumor of the left adrenal gland (*arrow*)

A 44-year-old Japanese man was referred to our hospital complaining of a large tumor that had been accidentally pointed out in a medical examination as being an esophageal submucosal tumor. Relevant family history brought to light that his father had been diagnosed as having MEN1. Our patient had undergone total parathyroidectomy and autologous transplantation for primary hyperparathyroidism at the age of 18. His height and weight were 187 cm and 120 kg, respectively, with a body mass index of 34 kg/m^2^ on admission. He also complained of sleep apnea and bilateral nipple discharge. He was not aware of the hypoglycemic symptoms before the admission, but he frequently encountered the symptoms under nutritional management after the admission. Laboratory data of blood chemistry were normal as well as the tumor markers, such as carcinoembryonic antigen and carbohydrate antigen 19-9, whereas neuron specific γ-enolase was high (19.1 ng/ml). Hormonal examinations revealed a hypoglycemia, a hyperinsulinemia (fasting serum glucose, insulin levels, and hemoglobin A1c, 76 mg/dl, 134.5 μU/ml, and 4.5 %, respectively), a hyperprolactinemia (serum prolactin level 871.2 ng/ml), and elevated urinary catecholamines. On an enhanced thin-slice abdominal computed tomography (CT), a large tumor was detected in the pancreatic tail, showing heterogeneous enhancement with partial calcification and cystic components. Its maximum diameter was about 18 cm (Fig.1a, b). Other than a pancreatic tail tumor, six enhanced liver tumors, a right adrenal tumor (3.9 cm in diameter), and a left adrenal tumor (2.6 cm in diameter) were found (Fig. 1c, d). The right adrenal tumor was heterogeneously enhanced. An endoscopic ultrasonography (EUS) revealed four tumors in the body and tail of the pancreas, except for the giant tumor, and abundant blood flow in all these tumors. Immunohistochemical examinations of a EUS-guided fine-needle aspiration biopsy showed that the giant pancreatic tumor and liver tumors were positive for chromogranin A, synaptophysin, and CD56; thus, the tumors were diagnosed as being a pancreatic neuroendocrine tumor (NET) and its liver metastases. They were NET G1 as the labeling index of Ki67 was 1.8 %. A calcium gluconate injection into the distal splenic artery during a selective intra-arterial calcium injection test resulted in a marked increase in serum insulin and C-peptide. Both ^123^I-metaiodobenzylguanidine scintigraphy and ^131^I-adosterol scintigraphy showed high radioactivity in the right adrenal tumor and no radioactivity in the left one. The left adrenal tumor was therefore diagnosed as having a non-functional adenoma. A magnetic resonance imaging (MRI) identified a heterogeneous pituitary adenoma, 3.1 cm in diameter.

**Fig. 2:**
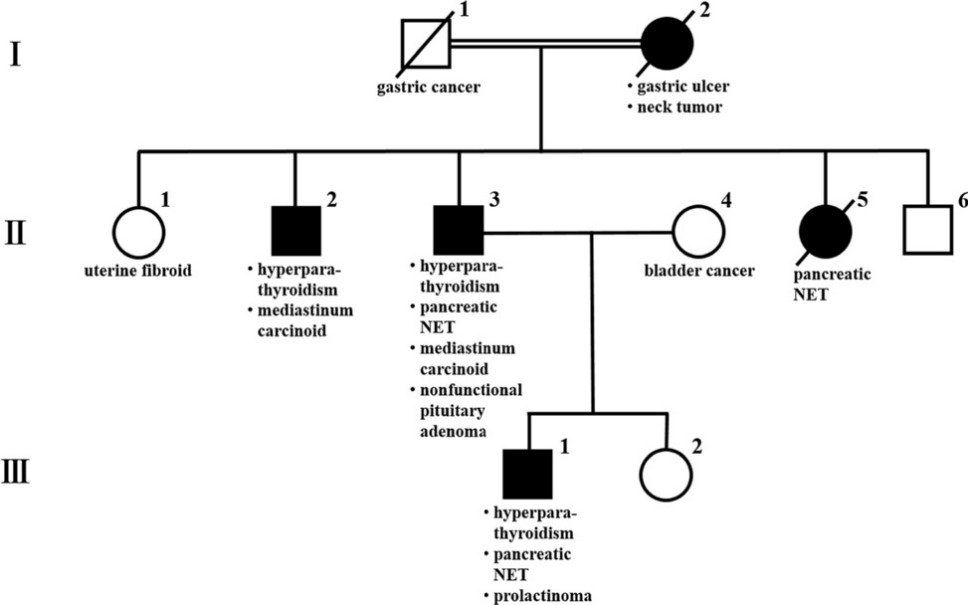
Family pedigree. This family pedigree was made according the standardized human pedigree nomenclature of the National Society of Genetic Counselors. *III-1* shows our patient. *I-2*, *II-1*, *II-2*, *II-3*, *II-5*, *II-6*, *III-1*, and *III-2* received the genetic examination. *NET* neuroendocrine tumor

**Fig. 3:**
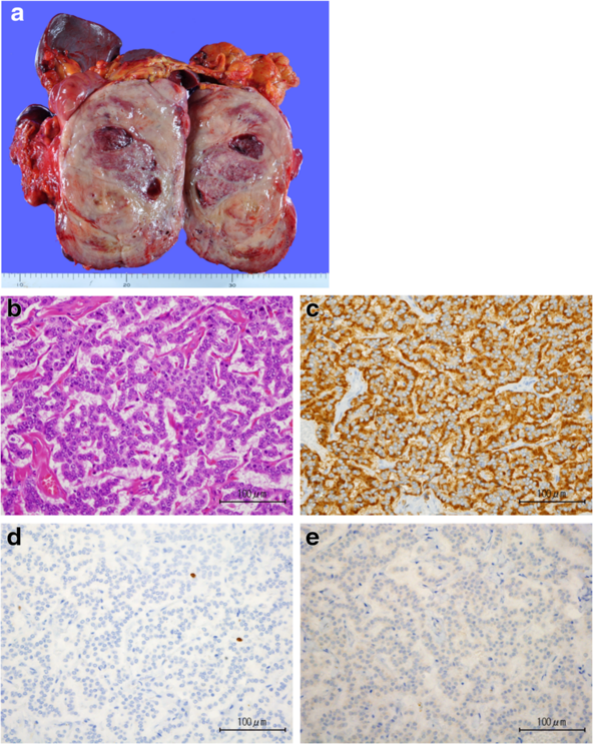
**a** Excised insulinoma, measuring 18.0 × 13.0 × 12.0 cm. **b** Hematoxylin-eosin staining of the insulinoma (×400). **c** Tumor cells are positive in the immunohistochemical staining for chromograin A (×400). **d** Ki67 is 4.3 % (×400). **e** Tumor cells are focally positive for insulin (×400)

**Fig. 4:**
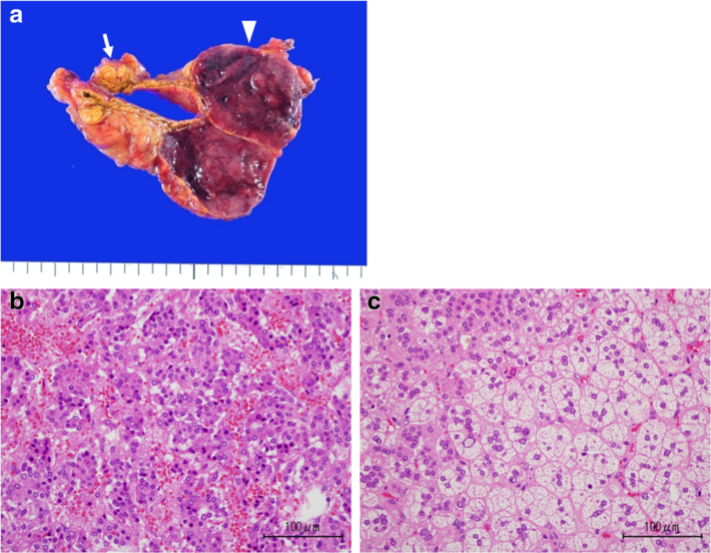
**a** The cut section of the excised right adrenal gland. The *arrow* showing the cortical adenoma and the *arrowhead* showing pheochromocytoma. **b** Hematoxylin-eosin staining of the pheochromocytoma (×400). **c** Hematoxylin-eosin staining of the adrenal cortical adenoma (×400)

His pedigree diagram is shown in Fig. 2. Five patients with MEN1, including our patient who has an *MEN1* gene mutation, were identified within his family. Furthermore, the genetic examination revealed a deletion mutation (g.249_252delGTCT) at codons 83–84 in exon 2. He underwent distal pancreatectomy, splenectomy, cholecystectomy, right adrenalectomy, abdominal subcutaneous tumor excision, and cutaneous tumor biopsy for the purpose of tumor volume reduction. Six tumors in the resected pancreas were diagnosed as being pancreatic NETs. The giant tumor in the pancreatic tail was insulinoma, and NET G2 as the Ki67 labeling index was 4.2 % (Fig. 3). Of the other five lesions, two tumors consisted of the insulin-positive tumor cells with less than 0.1 % of the Ki67 index, and three tumors consisted of the glucagon-positive tumor cells with less than 0.1 % of the Ki67 index. These tumors were diagnosed as being non-functional NET G1 because no symptom due to a glucagon excess was encountered. A non-functional adrenal cortical adenoma, measuring 1.3 cm in maximum diameter, and a pheochromocytoma, measuring 4.7 cm in maximum diameter, coexisted in the right adrenal gland (Fig. 4). The abdominal subcutaneous tumor was found to be a neurofibroma, and the cutaneous tumor was a fibroma.

During the postoperative outpatient follow-up, he developed diabetes mellitus and required treatment with an oral antidiabetic drug. For the treatment of his pituitary prolactinoma, cabergoline was administered. Octreotide therapy for the multiple liver metastases of the NETs began 2 months after the operation. After the ninth administration of the octreotide, three new lesions were detected in the liver on a CT scan. His general condition was judged to be tolerable to undergo liver resection, and the radiological examinations showed that there was no recurrence except the liver. Complete surgical resection of liver metastases was therefore achieved by an extended right posterior segmentectomy with partial hepatectomy of S2, S3, and S8. Immunohistochemical examination revealed that liver metastases were derived from a glucagon-positive tumor of the pancreatic NET. The Ki67 labeling index of these lesions ranged from 1.5 to 5.4 %.

Nineteen months after the hepatectomy, a CT scan revealed a small nodule in the S4 of the liver. Twenty-three months after the hepatectomy, gadoxetic acid- or gadolinium ethoxybenzyl diethylenetriamine pentaacetic acid-enhanced MRI demonstrated multiple liver tumors. We started everolimus therapy following a diagnosis of unresectable pancreatic NET recurrence. He is still alive after 4 years and 4 months after the initial surgery, although he is suffering from worsened diabetes mellitus, a pituitary prolactinoma that led to a hemorrhage into the pituitary tumor, and an increasing size of the left nonfunctional adrenal tumor.

### Discussion

MEN1 is an autosomal-dominant hereditary syndrome caused by germline mutations of the *MENIN* gene that predisposes the development of endocrine and non-endocrine tumors with variable penetrance [1–4, 6]. The most frequent tumors in MEN1 patients arise in the parathyroid glands, the pancreas, and the pituitary gland [4, 6, 7]. The incidence of pancreatic NETs is reported to be from 50 to 70 %. Insulinoma is one of the pancreatic NETs arising in the setting of MEN1. In Japan, the mean tumor size of pancreatic NETs was reported to be 3.03 cm at the time of diagnosis [5], whereas that of insulinoma was smaller than 2 cm in approximately 70 % of insulinoma patients, probably because insulinoma is a symptomatic disorder [5]. There are some previous reports on a giant insulinoma [8–12], and the insulinoma with a diameter of 18 cm in the present case is the second largest by referring to the previous reports. Moreover, only the present case did not complain of hypoglycemic symptoms, whereas all other patients with giant insulinoma presented such symptoms. The reasons why the present case showed no hypoglycemic symptoms were unclear; however, we can propose some hypotheses for this: pheochromocytoma might reduce hypoglycemia due to insulinoma; at the time of hypoglycemia, our patient overate unconsciously; and glucagon secreted from glucagon-positive tumor cells compensates hypoglycemia.

More than a half of the patients with a giant insulinoma reportedly had a liver metastasis at the time of diagnosis. Liver metastases of the present case were estimated to be derived from the glucagon-positive cells of the pancreatic NET. This was consistent with the consensus that non-functional tumor have a higher incidence of liver metastases than insulinoma [5]. On the other hand, the fact that the Ki67 labeling index of metastatic tumors was higher than that of the primary tumors suggested that highly proliferative components had more potential to form metastatic lesions.

An incidence of adrenal involvement in patients with MEN1 is about 20 % [4], most of which lesions are derived from the adrenal cortex. Concomitant pheochromocytoma is rare [6, 13]. Corticomedullary mixed tumor is a single adrenal tumor mass composed of an intimately admixed population of both adrenal cortical cells and pheochromocytes [14]. However, in our case, adrenal cortical adenoma and pheochromocytoma separately coexisted in the same adrenal gland, which is extremely rare [15–19], and even more so in the setting of the MEN1.

## Conclusions

In conclusion, we reported an extremely rare case of a giant insulinoma and simultaneous occurrence of pheochromocytoma and adrenal cortical adenoma in the ipsilateral adrenal gland in a patient clinically and genetically diagnosed as having MEN1.

## Consent

Written informed consent was obtained from the patient for publication of this case report and any accompanying images. A copy of the written consent form is available for review by the editor-in-chief of this journal.

## Abbreviations

MEN1: Multiple endocrine neoplasia type 1
NETs: Neuroendocrine tumors
CT: Computed tomography
EUS: Endoscopic ultrasonography
MRI: Magnetic resonance imaging
